# ‘Honeycomb appearance’ on three-dimensional transthoracic echocardiography as the landmark of left ventricular non-compaction: two case reports

**DOI:** 10.1186/1752-1947-7-142

**Published:** 2013-05-29

**Authors:** Takao Konishi, Tomoo Nagai, Akira Hamabe, Junko Arakawa, Hideki Hisadome, Mikoto Yoshida, Hirotsugu Tabata

**Affiliations:** 1 Department of Cardiology, Japan Self Defense Forces Central Hospital, Ikejiri 1-2-24, Setagaya-Ku, Tokyo 154-0001, Japan

**Keywords:** Left ventricular non-compaction, Magnetic resonance imaging, Three-dimensional echocardiography

## Abstract

**Introduction:**

Left ventricular non-compaction is a rare congenital heart disease, and is most commonly diagnosed via two-dimensional echocardiography according to echocardiographic criteria. Recently, transthoracic three-dimensional echocardiography has become available in the clinical setting.

**Case presentation:**

We present two isolated cases of left ventricular non-compaction from Japan (in an 84-year-old woman and 47-year-old man) that were confirmed by two-dimensional echocardiography, contrast-enhanced two-dimensional echocardiography, three-dimensional echocardiography and cardiac magnetic resonance imaging. In both cases, three-dimensional echocardiography successfully demonstrated the trabecular meshwork of the left ventricle, referred to as a ‘honeycomb appearance’.

**Conclusions:**

Three-dimensional echocardiography has the advantage of visualizing an en-face view of the trabecular meshwork, which is not possible with two-dimensional echocardiography. We further emphasize the clinical utility of three-dimensional echocardiography, which is not limited to just the observation of the trabeculations and inter-trabecular recesses, but can also visualize the trabecular meshwork with a ‘honeycomb appearance’.

## Introduction

Left ventricular non-compaction (LVNC) is a rare congenital heart disease that results from an arrest of the normal process of intra-uterine endomyocardial morphogenesis [1]. Chin *et al*. first described the typical morphologic features of LVNC, which are characterized by the persistence of numerous, deep trabeculations that communicate with the ventricular cavity as well as a trabecular meshwork pattern [2]. The clinical manifestations vary from the absence of symptoms to critical cardiac statuses such as heart failure, arrhythmias, and cardiogenic embolism. Patients with LVNC occasionally have evidence of familial disease, and significant genetic heterogeneity has been reported [3].

LVNC can be recognized by multiple imaging modalities, such as cardiac magnetic resonance imaging (CMR), two-dimensional echocardiography (2DE), contrast-enhanced 2DE, and angiography [4,5]. CMR, which provides a comprehensive depiction of cardiac morphology in any imaging plane, has been used to distinguish myocardial trabeculations from global LV mass [6]. LVNC is most commonly diagnosed by 2DE according to echocardiographic criteria; however, this method of diagnosis is still under much debate. Recently, transthoracic three-dimensional echocardiography (3DE) has become available in the clinical setting and is known to provide enhanced diagnostic capability [7].

In this report, we present two cases of isolated LVNC that were confirmed using 3DE by the typical trabecular meshwork of the left ventricle, referred to as a ‘honeycomb appearance’.

## Case presentation

### Case 1

**Figure 1:**
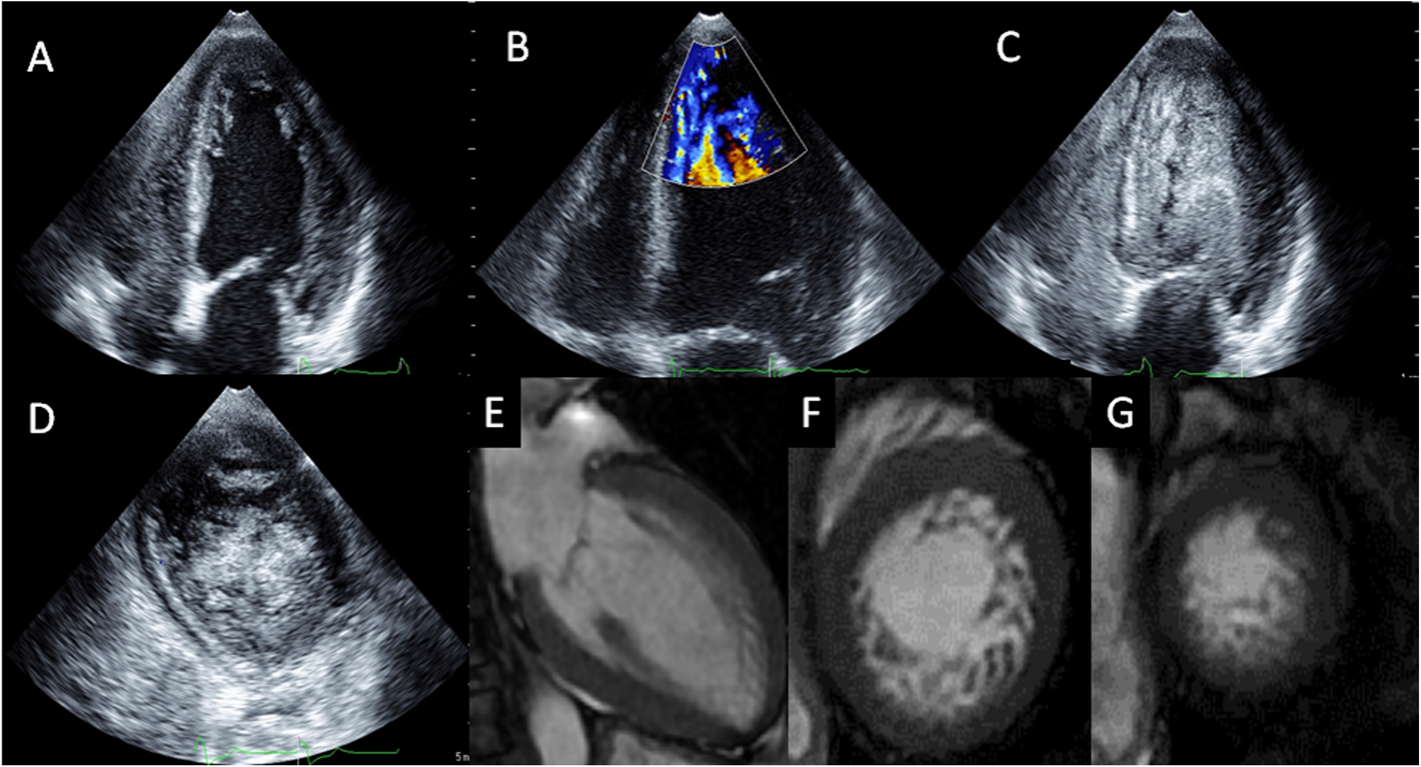
**(A) Two-dimensional echocardiogram showing marked trabeculations protruding from the left ventricular wall in the apex in our patient (Case 1). (B)** Blood perfusion of the inter-trabecular recesses from the left ventricular cavity observed by color Doppler study in Case 1. **(C,D)** Contrast-enhanced echocardiography confirmed the blood flow in the inter-trabecular recesses in Case 1. **(E-G)** Cardiac magnetic resonance imaging clearly visualized the boundary between the compaction layer and non-compaction layer in Case 1.

**Figure 2:**
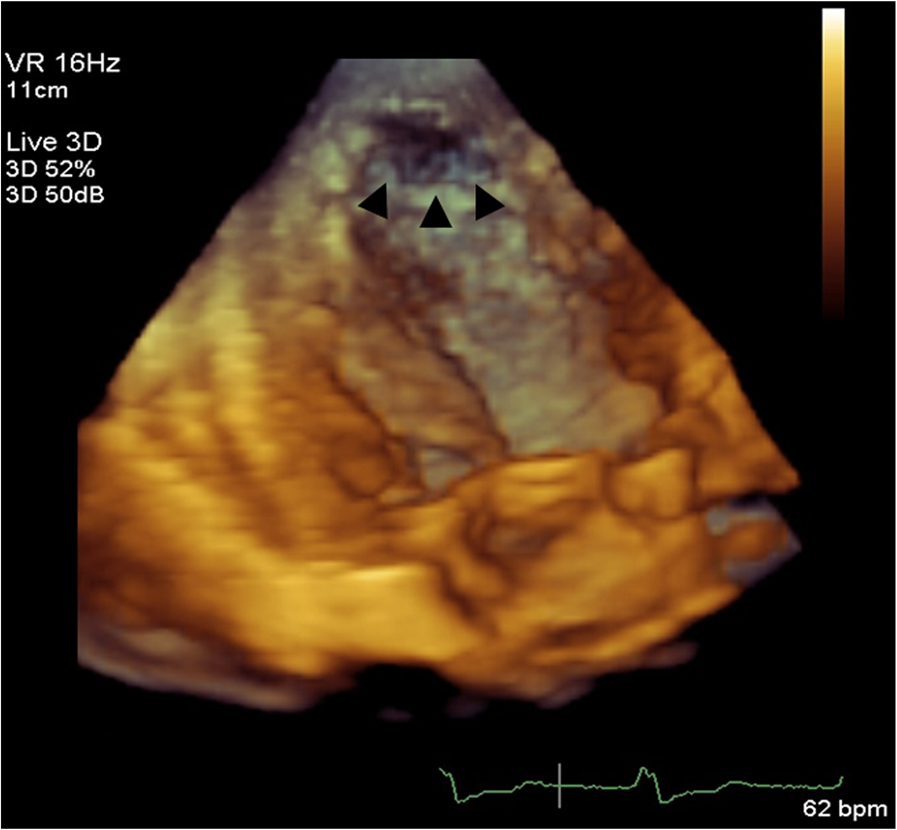
Real-time three-dimensional echocardiography demonstrates the typical ‘honeycomb appearance’ (arrowheads) in the apical lateral wall in our patient (Case 1).

An 84-year-old Japanese woman was admitted to our hospital with dyspnea. Her physical examination results indicated a pulse rate of 84 beats per minute, blood pressure of 124/72mmHg, fine crackles in both lungs, and pre-tibial edema in her legs. Her neurological examination results did not show neuromuscular abnormalities. Her electrocardiogram results showed normal sinus rhythm and a complete left bundle block. Laboratory study results showed mild anemia (hemoglobin, 10.8g/dL). Other basic laboratory data were normal. The results of a chest X-ray revealed pleural effusion in both lungs and cardiomegaly with a cardiothoracic ratio of 65 percent. 2DE performed in the emergency room showed mild to moderate mitral regurgitation, a normal size of left ventricle by 55mm, an enlargement of the left atrium by 47mm, and globally reduced left ventricular function with an ejection fraction of 26 percent. She received standard medical therapy for heart failure, which comprised intravenous and oral diuretics, a beta-adrenergic blocker, and an angiotensin-converting enzyme inhibitor. Once her condition stabilized, further medical investigation of the etiology was performed, including cardiac catheterization, contrast-enhanced 2DE, real-time 3DE, and CMR. Her coronary angiogram indicated severe stenosis in the proximal left anterior descending coronary artery and right coronary artery, which was successfully treated by percutaneous coronary angioplasty. Repeated 2DE showed marked trabeculations protruding from the left ventricular wall in the apex (Figure 1A). Blood perfusion of the inter-trabecular recesses from the left ventricular cavity was observed via color Doppler study (Figure 1B) and further confirmed by the use of intravenous echo contrast (Figure 1C,D). The ratio of the thickness of the non-compacted myocardial layer divided by the compacted myocardial layer at the end systole was 2.4, which met the Jenni echocardiographic criteria for LVNC [8]. Moreover, real-time 3DE demonstrated a ‘honeycomb appearance’ in the apical lateral wall (Figure 2). Finally, CMR was performed and showed typical left ventricular morphology of LVNC, although she refused the use of contrast agent (Figure 1E-G). Afterwards, familial screening by 2DE was performed and it was found that her daughter, son, and granddaughter also had massive trabeculations at the left ventricular apex. Thus, we concluded that LVNC could be the main cause of heart failure, although her acquired coronary artery disease and valvular heart disease may have contributed to the development of her condition.

### Case 2

**Figure 3:**
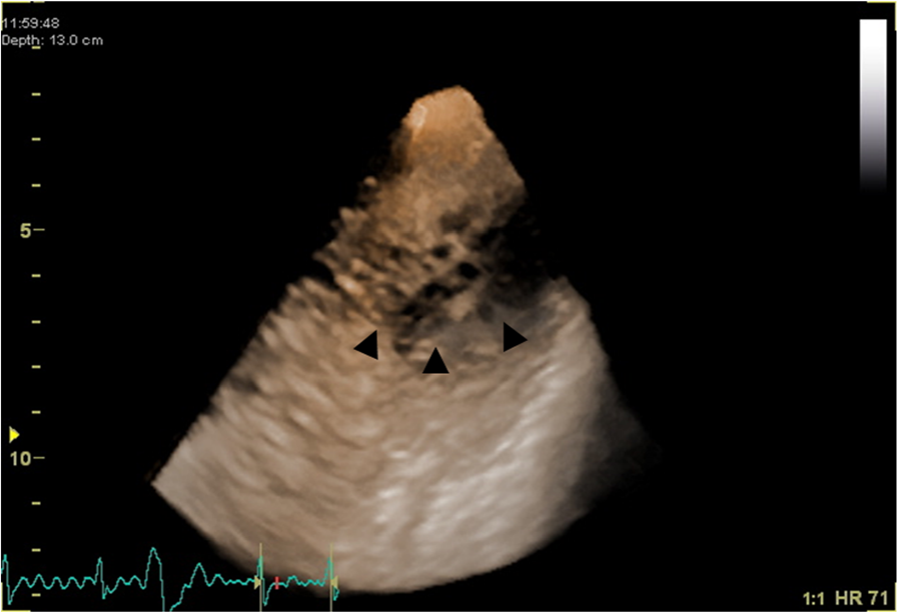
Real-time three-dimensional echocardiography demonstrated the ‘honeycomb appearance’ in the apical lateral wall (arrowheads) in our patient (Case 2).

A 47-year-old Japanese man with no history of heart disease was admitted to our hospital for medical investigation of an abnormal electrocardiogram of atrial premature contractions during an annual health check-up. His physical examination results revealed a pulse rate of 64 beats per minute, blood pressure of 134/80mmHg, normal respiratory sounds, and third heart sounds. His neurological examination results did not show any neuromuscular abnormalities. His electrocardiogram results showed normal sinus rhythm without conduction abnormalities, abnormal Q waves and ST-T changes. Laboratory test results were normal. The results of a chest X-ray revealed cardiomegaly with a cardiothoracic ratio of 58 percent. Holter monitoring revealed atrial and ventricular premature contractions. 2DE imaging showed mild mitral regurgitation, an enlargement of the left ventricle by 60mm, and globally reduced left ventricular function with an ejection fraction of 26 percent. Additionally, contrast-enhanced 2DE, real-time 3DE, myocardial perfusion imaging, CMR, and cardiac catheterization were performed. He was diagnosed as having LVNC on the basis of the findings derived from the above-mentioned imaging modalities. A ‘honeycomb appearance’ in the apex was observed on 3DE (Figure 3). He started taking oral medications including a beta-adrenergic blocker, an angiotensin-converting enzyme inhibitor and warfarin. One year later, he was hospitalized again due to congestive heart failure, and discharged with additional oral diuretics after intensive medical treatment lasting 18 days. 2DE was performed on his daughter, but no abnormalities were detected.

## Discussion

In the early embryo stage of human development, the heart is a loose, interwoven mesh of muscle fibers that form trabeculae with deep inter-trabecular recesses [9]. During the second month of embryonic life, the myocardium gradually condenses; compaction of the ventricular myocardium and transformation of the inter-trabecular spaces into capillaries occur with the development of coronary circulation [10]. This process of compaction occurs from the epicardium to the endocardium and from the base of the heart toward the apex [2]. In cases with LVNC, the process of compaction arrests at the early stage, and many prominent trabeculations and deep inter-trabecular recesses remain, primarily in the apex [11]. These lead to a segmental, trabecular meshwork in the left ventricle, which is a typical morphological feature of LVNC.

Recent studies have reported that the diagnosis of LVNC is often delayed because of difficulty revealing the diagnostic findings [1]. Multiple 2DE studies are required for a definite diagnosis.

3DE facilitates accurate diagnosis, detailed characterization, and description of the extent of the affected myocardium in LVNC. Entire trabeculations and inter-trabecular recesses are visualized and the boundary between the compact and non-compact myocardium is easily separated [12]. Moreover, Bodiwala *et al*. reported the usefulness of 3DE to visualize the trabecular meshwork, referred to as a ‘honeycomb appearance’, for the purpose of differentiating LVNC from other diseases such as right ventricular dysplasia [13]. However, to the best of our knowledge, only a limited number of case reports have mentioned this unique feature of LVNC on 3DE [14]. In both of our patients featured in the present report, we could clearly document the ‘honeycomb appearance’ in the apex, and easily make a precise diagnosis of LVNC that was supported by contrast-enhanced 2DE and CMR.

## Conclusions

3DE has the advantage of visualizing an en-face view of the trabecular meshwork, which is not possible by 2DE. We further emphasize the clinical utility of 3DE, which is not limited to the observation of the trabeculations and inter-trabecular recesses, but also the trabecular meshwork with a ‘honeycomb appearance’.

## Consent

Written informed consents were obtained from the patients for publication of this manuscript and any accompanying images. A copy of the written consents are available for review by the Editor-in-Chief of this journal.

## Abbreviations

2DE: two-dimensional echocardiography; 3DE: three-dimensional echocardiography, LVNC, left ventricular non-compaction; MRI: magnetic resonance imaging.

## Competing interests

The authors declare that they have no competing interests.

## Authors’ contributions

TK and TN wrote and revised the manuscript. AH and JA gave support in performing the cardiac magnetic resonance imaging. HH and MY gave support in performing the three-dimensional echocardiography and the contrast echocardiography. HT gave final approval of the version to be published. All authors read and approved the final manuscript.
